# Covalency does not suppress O_2_ formation in 4d and 5d Li-rich O-redox cathodes

**DOI:** 10.1038/s41467-021-23154-4

**Published:** 2021-05-20

**Authors:** Robert A. House, John-Joseph Marie, Joohyuk Park, Gregory J. Rees, Stefano Agrestini, Abhishek Nag, Mirian Garcia-Fernandez, Ke-Jin Zhou, Peter G. Bruce

**Affiliations:** 1 Department of Materials and Chemistry, University of Oxford, Oxford, UK; 2 The Henry Royce Institute, Oxford, UK; 3 The Faraday Institution, Quad One, Becquerel Avenue, Harwell Campus, Didcot, UK; 4 Diamond Light Source, Harwell Campus, Didcot, UK

**Keywords:** Electronic materials, Batteries, Batteries

## Abstract

Layered Li-rich transition metal oxides undergo O-redox, involving the oxidation of the O^2−^ ions charge compensated by extraction of Li^+^ ions. Recent results have shown that for 3d transition metal oxides the oxidized O^2−^ forms molecular O_2_ trapped in the bulk particles. Other forms of oxidised O^2−^ such as O_2_^2−^ or (O–O)^n−^ with long bonds have been proposed, based especially on work on 4 and 5d transition metal oxides, where TM–O bonding is more covalent. Here, we show, using high resolution RIXS that molecular O_2_ is formed in the bulk particles on O^2‒^ oxidation in the archetypal Li-rich ruthenates and iridate compounds, Li_2_RuO_3_, Li_2_Ru_0.5_Sn_0.5_O_3_ and Li_2_Ir_0.5_Sn_0.5_O_3_. The results indicate that O-redox occurs across 3, 4, and 5d transition metal oxides, forming O_2_, i.e. the greater covalency of the 4d and 5d compounds still favours O_2_. RIXS and XAS data for Li_2_IrO_3_ are consistent with a charge compensation mechanism associated primarily with Ir redox up to and beyond the 5+ oxidation state, with no evidence of O–O dimerization.

## Abstract (web summary)

In this work, authors show that O-redox in 4d and 5d transition metal oxides involves the formation of molecular oxygen trapped in the particles. These results are in accord with observations in 3d oxides and show that the greater covalency of the 4d and 5d oxides does not stabilise peroxo-like species.

## Introduction

Layered Li-rich 3d transition metal oxide intercalation compounds, such as Li[Li_0.2_Ni_0.13_Co_0.13_Mn_0.54_]O_2_, have received a great deal of attention because Li^+^ can be extracted beyond the limit of transition metal (TM) oxidation, with the charge being compensated by oxidation of the O^2‒^ ions (O-redox)^1–9^. These compounds typically possess honeycomb ordered Li and TM within the TM layer. However, Mn-based honeycomb ordered structures do not provide a stable framework for oxidised O^2−^ and have been shown to undergo extensive TM migration, and bulk O–O dimerization leading to voltage hysteresis and loss of energy density, in addition to surface O_2_ evolution^10–14^. It has been shown recently that the dimerised O–O is molecular O_2_, which is trapped in voids within the bulk of the charged particles^15,16^. Molecular O_2_ formation is responsible for both surface O-loss and bulk O-redox.

Pioneering work by Tarascon, Doublet and co-workers^17–25^ and by others^26–31^, on the 4d- and 5d-based analogues of the 3d compounds, such as Li_2_RuO_3_, Na_2_RuO_3_, Li_2_Ru_0.5_Mn_0.5_O_3_, Li_2_IrO_3_ and Li_2_Ir_0.75_Sn_0.25_O_3_, has led to important advances in the understanding of O-redox. These systems possess the same honeycomb ordered Li and TM ions within the TM layer. With the exception of Li_2_IrO_3_, they exhibit voltage hysteresis, with a plateau on charge and a low voltage S-shaped profile on subsequent discharge. Loss of honeycomb ordering due to Li/TM disordering accompanies the voltage hysteresis along with O_2_ loss from the surface of the particles^17,19,21,26,29^. It has been reported that peroxides O_2_^2−^ and longer O–O dimers form beyond the limits of transition metal oxidation in the 4 and 5d transition metal oxides^17,19^. The more strongly hybridised TM–O bonding of the 4 and 5d transition metals compared with the 3d counterparts has been cited as a reason for stabilising such O–O species with longer O–O bonds^18–20,32^. In highly covalent transition metal sulphides, selenides and tellurides, electron holes can be stabilised through dimerization of the chalcogen (S_2_)^2−^ which remains coordinated to the transition metal due to the strong orbital overlap^33^.

It has proved very challenging to identify experimentally the form of oxidised O^2−^ in charged materials. The recent application of high resolution RIXS spectroscopy has proved useful in probing the nature of oxidised O^2−^^15,16^. Here we apply this technique to the 4 and 5d materials, providing direct evidence for the presence of molecular O_2_, trapped in the bulk of the archetypal 4d and 5d systems Li_2_RuO_3_, Li_2_Ru_0.5_Sn_0.5_O_3_ and Li_2_Ir_0.5_Sn_0.5_O_3_. The O_2_ is formed only through charging across the high voltage plateau and is reduced on subsequent discharge, in line with our findings for Li_1.2_Ni_0.13_Co_0.13_Mn_0.54_O_2_, disordered rocksalt Li_2_MnO_2_F and the P2-type Na-ion cathodes^15,16,34^. We find no evidence of molecular O_2_ formation or other O–O dimers in Li_2_IrO_3_ up to the limits of oxidation and instead identify stable electron holes formed in bonding TM–O orbitals, consistent with previous reports indicating reversible Ir^5+^ to Ir^5.5+^ oxidation instead of O oxidation and reinforcing the link between bulk O_2_ formation and the loss of high voltage plateau^26^. These data indicate that the more covalent TM–O bonding in 4 and 5d compared with 3d TM oxides still favours the formation of molecular O_2_, helping to explain why O-loss is also observed from the surface of these compounds. The implication is that the O-redox process, involving molecular O_2_ formation at the surface and in the bulk, is the same for Li-rich systems with the honeycomb superstructure moving down the Periodic Table.

## Results

### The Li-rich ruthenates and iridates

**Fig. 1:**
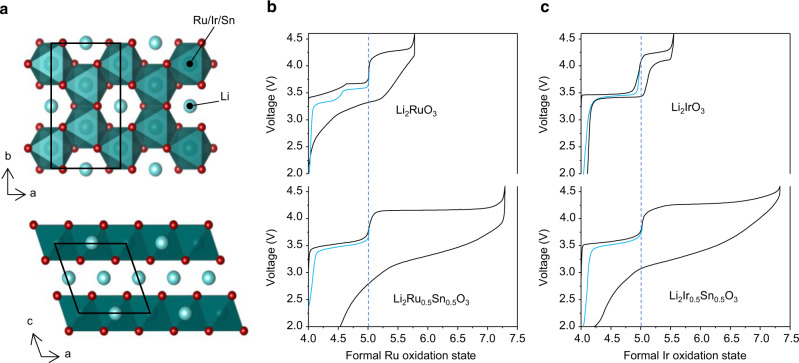
Structure and electrochemistry of Li-rich 4d- and 5d-based cathodes. **a** O3-type layered structure with honeycomb ordering of Li and TM ions within the TM layers. First cycle load curves for **b** Li_2_RuO_3_ and Li_2_Ru_0.5_Sn_0.5_O_3_ and (**c**) Li_2_IrO_3_ and Li_2_Ir_0.5_Sn_0.5_O_3_ from 2.0 V to 4.6 V at a current rate of 20 mA g^−1^ plotted against formal oxidation state of Ru or Ir (in black). Electrochemical cycling is reversible with little voltage hysteresis below +5 (in blue). High voltage charging plateaus are observed in all materials beyond the +5 TM oxidation state. The plateaus are accompanied by significant voltage loss on subsequent discharge in all cases except for Li_2_IrO_3_.

Li_2_RuO_3_, Li_2_IrO_3_ and Sn-substituted Li_2_Ru_0.5_Sn_0.5_O_3_ and Li_2_Ir_0.5_Sn_0.5_O_3_ were prepared following the methods of previous reports^17,19^. Powder X-ray Diffraction data, Supplementary Figs. 1–3, confirm the formation of the compounds. Each of the materials possess O3-type layered structures with honeycomb ordering in the TM plane, Fig. 1a manifesting as the familiar superlattice peaks between 2θ = 18° and 34°. As seen before, there is evidence of some stacking faults between the ordered layers, which result in asymmetric peak broadening of these superlattice peaks, especially for the Sn-substituted samples.

The first cycle load curves are shown in Fig. 1b, c and are plotted against the nominal oxidation state of Ru and Ir in each case, since Sn^4+^ is known to be redox inactive. The electrochemical behaviour is reversible with very little hysteresis when cycling below Ru^5+^ and Ir^5+^. When sufficient Li is extracted to exceed the +5 oxidation state on Ru, an extended high voltage plateau is observed followed by an S-shaped discharge. For the iridates, reversibility can be maintained up to +5.5 supported by Ir redox as shown recently by Hong et al.^26^. Further Li can be extracted beyond this limit in Li_2_Ir_0.5_Sn_0.5_O_3_ subsequently inducing voltage hysteresis. Li_2_IrO_3_ is the only material where TM migration and loss of the honeycomb ordering is avoided upon charging to 4.6 V, in accord with its reversible electrochemical behaviour^26,30^.

### Spectroscopic characterisation of O

Understanding O-redox has proven to be a challenge due in part to the need for techniques capable of determining the nature of O species formed in the bulk. In this study, we have employed X-ray absorption spectroscopy (XAS) in partial fluorescence yield (PFY) mode and high resolution resonant inelastic X-ray scattering (RIXS) at the O K-edge, as they offer a direct probe of the electronic states on O at depths of up to 50–100 nm into the sample. XAS probes the empty states above the Fermi level. In RIXS, excitation of core electrons to empty states above the Fermi level results in emission as electrons from filled valance states below the Fermi level relax to the core-hole states. RIXS complements XAS as it provides a direct probe of the valence states on O.

**Fig. 2:**
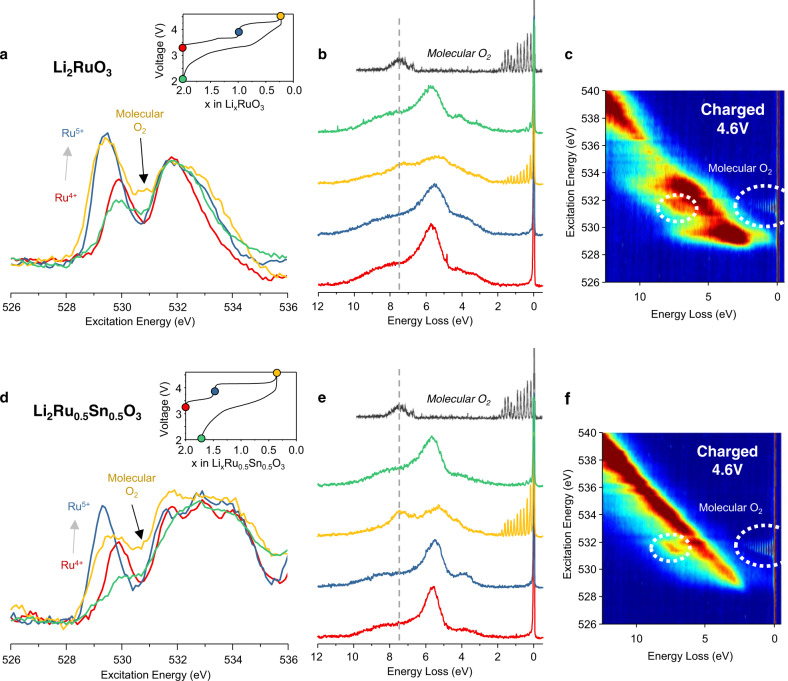
O XAS and RIXS spectroscopy for Li-rich ruthenates. **a**, **d** O K-edge XAS spectra collected in bulk sensitive partial fluorescence yield (PFY) mode. Inset figure—load curve showing with coloured dots where on the load curve the spectra were collected. **b**, **e** high resolution RIXS line scans at an excitation energy of 531 eV. Spectrum of molecular O_2_ gas shown for reference in black (reproduced from ref. ^35^). The RIXS line scans reveal the formation of molecular O_2_ which is reduced to O^2−^ on discharge. **c**, **f** High resolution RIXS maps of the O K-edge pre-edge for cathodes charged to the end of the high voltage plateau. Across the plateau there is an increase in area at 531 eV for both Li_2_RuO_3_ and Li_2_Ru_0.5_Sn_0.5_O_3_. The RIXS maps show that there are no other O–O vibrations.

In Fig. 2, we present the O K-edge XAS and RIXS for Li_2_RuO_3_ and Li_2_Ru_0.5_Sn_0.5_O_3_ collected ex situ at different points along the load curve on charge and discharge. Considering first the XAS spectra. For Li_2_RuO_3_, on initial charge to the beginning of the plateau, there is a pronounced increase in intensity at the leading edge of the pre-edge (lowest energy peak between 529 and 530 eV) indicating the formation of electron hole states in hybridised Ru–O orbitals consistent with Ru oxidation from +4 to +5, as previously reported^17,25^. Across the plateau there is no further increase in this region but instead new states appear at 531 eV. After discharge, both of these changes are reversed, and the pre-edge reduces in intensity. The pre-edge for the discharged sample is comparatively broad when compared directly with that of the pristine indicating a rehybridization of the Ru–O bonding between the two samples. The structure has been shown to undergo TM migration during the first cycle and the XAS peak broadening we observe here is consistent with an increase in the local disorder around O. For Li_2_Ru_0.5_Sn_0.5_O_3_, the pre-edge broadening after the plateau on charge and in the discharged material is more pronounced than Li_2_RuO_3_ in line with the greater degree of O-redox and more extensive TM migration for the Sn-substituted material^17^.

To interrogate the electronic states formed at 531 eV further, RIXS measurements were performed for each sample at this excitation energy. The emission spectra are plotted as is convention, as energy loss (difference between excitation and emission energy). At the top of charge two new energy loss features become evident, a broad peak at 8 eV and a progression of sharp peaks between 0 and 2 eV, as we observed previously for Li_1.2_Ni_0.13_Co_0.13_Mn_0.54_O_2_ and Na_0.75_Li_0.25_Mn_0.75_O_2_^15,16^. The progression of peaks in the 0–2 eV region correspond to the vibrations of a molecular O_2_ diatomic, also shown in Fig. 2^35^. The emission spectra for a range of excitation energies across the O K-edge for the fully charged electrodes were also measured and are presented as RIXS maps. The data show no evidence of any other vibrational features at different excitation energies. After discharge, these new features are much diminished in intensity indicating reversible electrochemical reduction of molecular O_2_ has occurred.

**Fig. 3:**
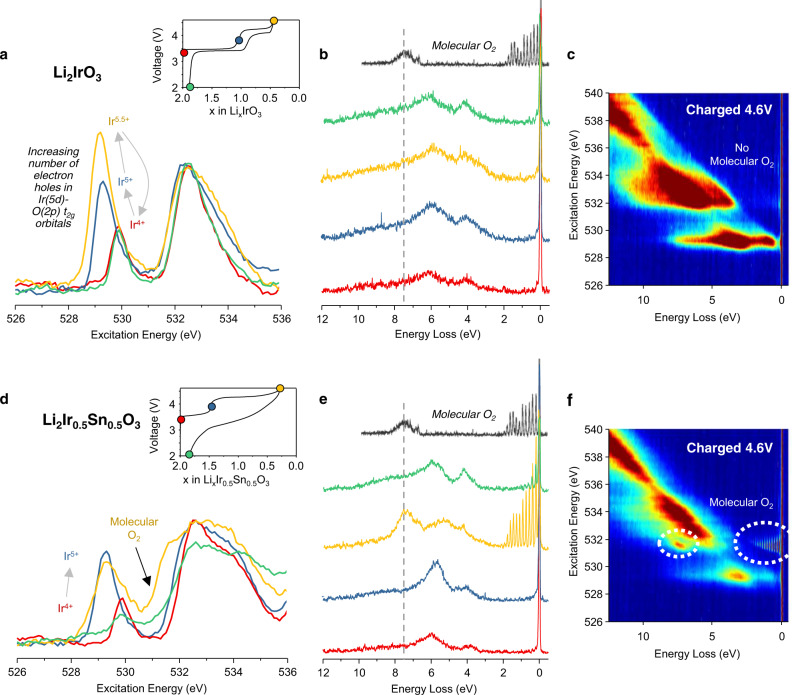
O XAS and RIXS spectroscopy of Li-rich iridates. **a**, **d** O K-edge XAS spectra collected in bulk sensitive partial fluorescence yield (PFY) mode. Inset figure—load curve showing with coloured dots where on the load curve the spectra were collected. **b**, **e** High resolution RIXS line scans at an excitation energy of 531 eV. Spectrum of molecular O_2_ gas shown for reference in black (reproduced from ref. ^35^). The RIXS line scans reveal the formation of molecular O_2_ in Li_2_Ir_0.5_Sn_0.5_O_3_ which is reduced to O^2−^ on discharge but not in Li_2_IrO_3_. **c**, **f** High resolution RIXS maps of the O K-edge pre-edge for cathodes charged to the end of the high voltage plateau. The RIXS maps show that there are no other vibrations.

The same measurements were also performed for the iridate samples and are presented in Fig. 3. Li_2_Ir_0.5_Sn_0.5_O_3_ exhibits very similar changes to those described for Li_2_RuO_3_ and Li_2_Ru_0.5_Sn_0.5_O_3_ consistent with the voltage profile observed. On the other hand, the RIXS spectra for Li_2_IrO_3_ do not show any evidence for the presence of molecular O_2_ in the fully charged electrodes. Instead, a strong increase in intensity at the leading edge of the pre-edge is seen when charging Ir beyond the +5 oxidation state. This observation supports the conclusion that the high voltage plateau in Li_2_IrO_3_ is associated with Ir rather than O oxidation^26^. Notably, the XAS spectra for the pristine and discharged electrodes are almost fully superimposable indicating minimal irreversible change to the electronic structure and thus structural stability.

Since the measurements are performed under ultra-high vacuum (UHV) conditions and the samples had been pumped down overnight under UHV, the electrodes will be fully out-gassed, so any molecular species that are detected are trapped within the bulk of the primary particles. To rule out the possible influence of beam damage inducing molecular O_2_, we performed all our measurements at low temperature, 20 K, and conducted measurements at the same sample location over a range of timescales. The data presented in Supplementary Fig. 4 show no change in the peak spacing between spectra acquired after 30 s and 1800 s exposure times and only a minor decrease in intensity, which is in line with our previous beam sensitivity studies for molecular O_2_ in Li_1.2_Ni_0.13_Co_0.13_Mn_0.54_O_2_^15^. In that paper, we also extensively examined the effect of temperature and photon flux and showed that neither of these factors have a detectable influence on the vibrational peak spacing, reinforcing that the O_2_ observed by RIXS is intrinsic to the cathode.

**Fig. 4:**
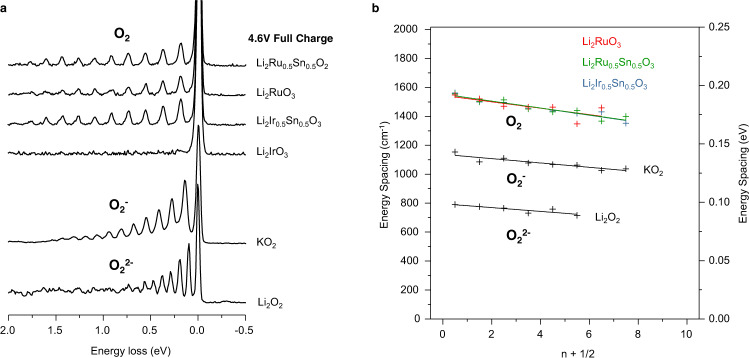
Direct spectroscopic evidence against peroxides in charged 4d and 5d cathodes. **a** High resolution RIXS scans at 531 eV excitation energy for charged cathode samples and a Li_2_O_2_ reference collected under identical measurement conditions. KO_2_ RIXS was collected at 529.6 eV, where O_2_^−^ vibrations are strongest. Peak spacing consistent with molecular O_2_ is seen in the case of all cathodes except Li_2_IrO_3_ which is not O-redox active. There is no evidence of O_2_^2−^ or O_2_^−^ vibrations in any of the cathode samples ruling out the presence of peroxides and superoxides. **b** Birge-sponer plot showing linearly decreasing dependence of peak spacing with vibrational quantum number, *n*, consistent with the anharmonic oscillations of diatomic molecules.

Although the spectroscopic data show no evidence of O species other than O_2_, RIXS data were also collected for KO_2_ and Li_2_O_2_ to rule out the possibility of O^2−^ and O_2_^2−^. The results are presented in Fig. 4 where they are compared directly with the spectra for the Ru and Ir compounds. The peak spacing for Li_2_O_2_ around the elastic peak corresponding to the vibrational spectrum is almost exactly half of the peak spacing observed for molecular O_2_ in the cathodes as clearly seen in the Birge-Sponer plot, Fig. 4b. This is closely in line with the vibrational frequency for O_2_^2−^ which is well known to be half that of molecular O_2_. For KO_2_, containing the superoxide moiety O_2_^−^ of intermediate bond order to O_2_ and O_2_^2−^, the peak spacing lies halfway between the two. While some differences in the RIXS spectra for different peroxide and superoxide compounds is possible, the vibrational spectra associated with the elastic peak is determined primarily by the O–O bond length/strength and therefore is characteristic of these species in general. The clear distinction that can be made between O_2_, O_2_^−^ and O_2_^2−^ dimers demonstrates the power of high resolution RIXS and provides evidence for the formation of molecular O_2_ in the Li-rich cathodes.

## Discussion

Studies using X-ray photoelectron spectroscopy (XPS), electron paramagnetic resonance (EPR) and density functional theory (DFT) pointed to the possibility of peroxo-like O_2_^n−^ species, where n = 1, 2 or 3^17,20,25,36^. Scanning transmission spectroscopy (STEM) and neutron powder diffraction studies of Li_2_IrO_3_, suggested longer peroxo-like dimers (3 < n < 3.3)^19^. However, excellent though these studies are, it is a challenge for these techniques to identify unambiguously the nature of the oxidised oxygen species. XPS, being an electron emission technique, is, in general, more limited in its ability to measure bulk species than RIXS, which utilises photon emissions, and XPS can often be strongly influenced by surface contributions. Turning to local structural probes, imaging individual O–O defects in the bulk is beyond the capabilities of current STEM techniques and resolving O–O species at such low interatomic separations and concentrations is very challenging for total scattering data. Since they are magnetically complex materials, the 4d and 5d systems defy clear characterisation of oxidised O by either ^17^O NMR or SQUID. In contrast, the high resolution RIXS that we employ in this study is element specific, probes 50–100 nm deep into the particles, and has allowed us to clearly identify molecular O_2_ in the bulk of solid materials. High resolution RIXS has already provided evidence that O^2−^ oxidation in 3d TM oxides forms molecular O_2_ trapped in voids in the bulk particles. This observation is further supported by ^17^O NMR which not only identifies trapped molecular O_2_ as the O–O species formed on charge, but also shows that it is present in quantities commensurate with that expected from the charged passed in Li_1.2_Ni_0.13_Co_0.13_Mn_0.54_O_2_^15^.

Our RIXS and XAS results indicate that the O-redox process can be described as molecular O_2_ formation throughout the cathode, both as evolved O_2_ at the surface, which has already been demonstrated with operando mass spec^19,29^, and trapped O_2_ within the bulk. The 4 and 5d transition metal oxides generally exhibit greater covalency in the TM–O bond than those of their 3d counterparts; associated with the greater TMd-O2p overlap and lower electron repulsion of the larger 4 and 5d orbitals. The results presented here indicate that these more covalent systems bear closer resemblance to the 3d Li-rich materials than previously thought and that any greater covalency in the TM–O bond for 4 and 5d compounds does not suppress molecular O_2_ formation in favour of other O–O species.

**Fig. 5:**
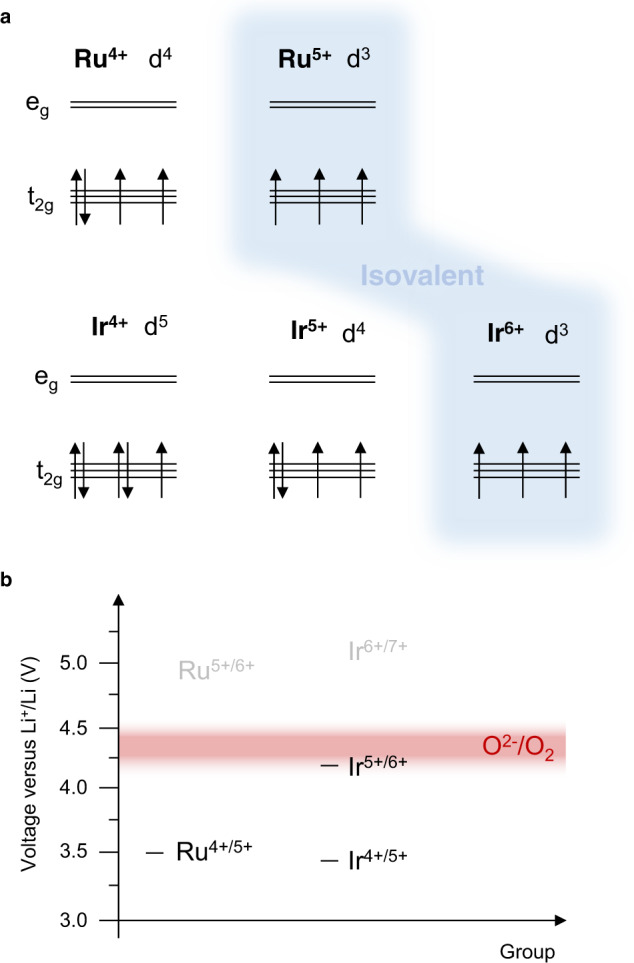
Redox potentials. **a** d electron configurations for different oxidation states of Ru and Ir. The spin down electron on Ir^5+^ is at a higher energy and hence oxidised at a lower voltage than removal of a spin up electron from the stable d^3^ configuration of Ru^5+^. Since oxidation of Ir^5+^ occurs before O^2−^/O_2_ and Ru^5+^ does not, we place O^2−^/O_2_ between the two redox couples in the figure (**b**). The values of the redox couples shown in black in (**b**) were obtained from dQ/dV analysis extracted from the data in Fig. 1.

In a recent study, Hong et al. presented Ir L_3_ XANES data that showed Ir is able to oxidise beyond +5 to +5.5 in Li_2_IrO_3_ and Li_2_Ir_0.5_Sn_0.5_O_3_ before O-redox activity^26^. In this regime, both materials can cycle reversibly. For Li_2_Ir_0.5_Sn_0.5_O_3_, charging can be continued beyond +5.5 consequently incurring O oxidation, TM migration, peroxide (O_2_^2−^) formation and voltage hysteresis. Our results show an absence of any oxidised oxygen species in Li_2_IrO_3_ in accord with the Ir L_3_ XANES data presented by Hong et al^26^. However, for Li_2_Ir_0.5_Sn_0.5_O_3_ the high resolution RIXS shows the presence of molecular O_2_, rather than peroxide, as the only form of oxidised oxygen species. High resolution RIXS also reveals molecular O_2_ is present in the ruthenates in contrast to previous reports of peroxides. The ability of RIXS to show an absence of signal for materials supported exclusively by TM-redox and identify oxidised O when it is present in O-redox materials demonstrates its utility for probing oxidised O species. Ir^5+^, low spin t_2g_^4^, has 1 more electron than Ru^5+^, t_2g_^3^, and it is spin-paired, Fig. 5a. Removal of this higher energy spin down electron occurs at a lower voltage than for an electron on Ru^5+^. The oxidation of O^2−^ sits between the energies for Ir^5+/6+^ and Ru^5+/6+^ such that Ir^5+^ is oxidised before O^2−^ (i.e. at a lower voltage) whereas Ru^5+^ is not. The voltages of the redox couples derived from dQ/dV analysis of the electrochemical load curves in Fig. 1 are shown in Fig. 5b. The substitution of Ir for Sn limits the Ir redox capacity increasing the amount of extractable Li available to be charge compensated by O-redox explaining why substantial O oxidation is observed for Li_2_Ir_0.5_Sn_0.5_O_3_.

Much like the 3d TM Li-rich O-redox systems, those based on 4d and 5d TM elements exhibit TM migration, loss of honeycomb ordering, O-loss and voltage hysteresis. The vibrational spectra measured by RIXS now also show that molecular O_2_, rather than peroxides or peroxo-like species, are formed in all of these systems indicating TM–O covalency has limited effect on the bond order of the O–O dimer in the bulk of the cathodes. Future research efforts on Li-rich cathodes should focus on chemical and structural modifications other than covalency of the host network to improve their performance.

## Methods

### Materials preparation

Li_2_RuO_3_ and Li_2_Ru_0.5_Sn_0.5_O_3_ were synthesised from RuO_2_ (99.9% Alfa Aesar), SnC_2_O_4_ (98% Alfa Aesar) and Li_2_CO_3_ (99+% Merck) mixed in the appropriate ratios with 10% excess Li_2_CO_3_. Calcination was performed in air at 800 °C for 6 h, 900 °C for 12 h and then 1100 °C for 12 h with intermediate grinding. Li_2_IrO_3_ and Li_2_Ir_0.5_Sn_0.5_O_3_ were synthesised from IrO_2_ (99.9% Alfa Aesar), SnO_2_ (99.9% Alfa Aesar) and Li_2_CO_3_ (99+% Merck) mixed in the appropriate ratios with 10% excess Li_2_CO_3_. Calcination was performed in air at 1000 °C for 12 h and 900 °C for 36 h with intermediate grinding. The as-prepared materials were transferred to an Ar-filled glovebox and handled under inert atmosphere for all further manipulations. Li_2_O_2_ (95%, ACROS Organics) and KO_2_ (Sigma Aldrich) standards were used as supplied.

### Electrochemistry

Self-supporting electrode films were prepared by grinding the as-synthesised materials with acetylene black and polytetrafluoroethylene in a 8:1:1 mass ratio in a pestle and mortar and subsequently calendared. Electrochemical cycling was performed in coin cells with LP30 electrolyte and a lithium metal foil counter electrode. Cells were disassembled at different states of charge and the electrodes rinsed with dry dimethylcarbonate for ex situ analysis.

### Powder X-ray diffraction

Powder X-ray diffraction patterns were obtained for the as-prepared materials using a Cu source Rigaku SmartLab diffractometer equipped with a Ge(220) double bounce monochromator and without exposure to air. Reitveld profile refinements were performed using the GSAS suite of programs.

### X-ray absorption spectroscopy and resonant inelastic X-ray scattering

X-ray absorption spectroscopy and resonant inelastic X-ray scattering data were obtained at the I21 beamline, Diamond Light Source. Samples were transferred to the spectrometer using a vacuum transfer suitcase to avoid air exposure and were pumped down to UHV and left to fully degas overnight. O K-edge spectra were obtained in partial fluorescence mode for bulk sensitivity. RIXS line scans were recorded at five different sample locations and averaged together. RIXS maps were collected at 0.2 eV intervals in excitation energy. All measurements were performed at 20 K to minimise any possible beam damage.

## Supplementary information

Supplementary Information

## Data Availability

Supporting research data has been deposited in the Oxford Research Archive and is available under this DOI: 10.5287/bodleian:eyyG8ovA0.
