# Supplementary material for: Covalency does not suppress O2 formation in 4d and 5d Li-rich O-redox cathodes
Source: Nat Commun. 2021 May 20;12:2975. doi: 10.1038/s41467-021-23154-4 (PMC8137948; doi:10.1038/s41467-021-23154-4)
Supplement: Supplementary file 1 — Supplementary Information [file 41467_2021_23154_MOESM1_ESM.pdf]

Supplementary Information

Covalency does not suppress O_2 formation in 4d and 5d Li-rich O-redox cathodes

Robert A. House[1,2,3], John-Joseph Marie[1,2,3], Joohyuk Park[1,2,3], Gregory J. Rees[1,2,3], Stefano Agrestini[4], Abhishek Nag[4], Mirian Garcia-Fernandez[4], Ke-Jin Zhou[4], Peter G. Bruce[1,2,3*]

1. Department of Materials and Chemistry, University of Oxford, Parks Road, Oxford, OX1 3PH, U.K.
2. The Henry Royce Institute, Parks Road, Oxford, OX1 3PH, U.K.
3. The Faraday Institution, Quad One, Becquerel Avenue, Harwell Campus, Didcot, OX11 0RA, U.K.
4. Diamond Light Source, Harwell Campus, Didcot, U.K.

[*]Corresponding Author: peter.bruce@materials.ox.ac.uk

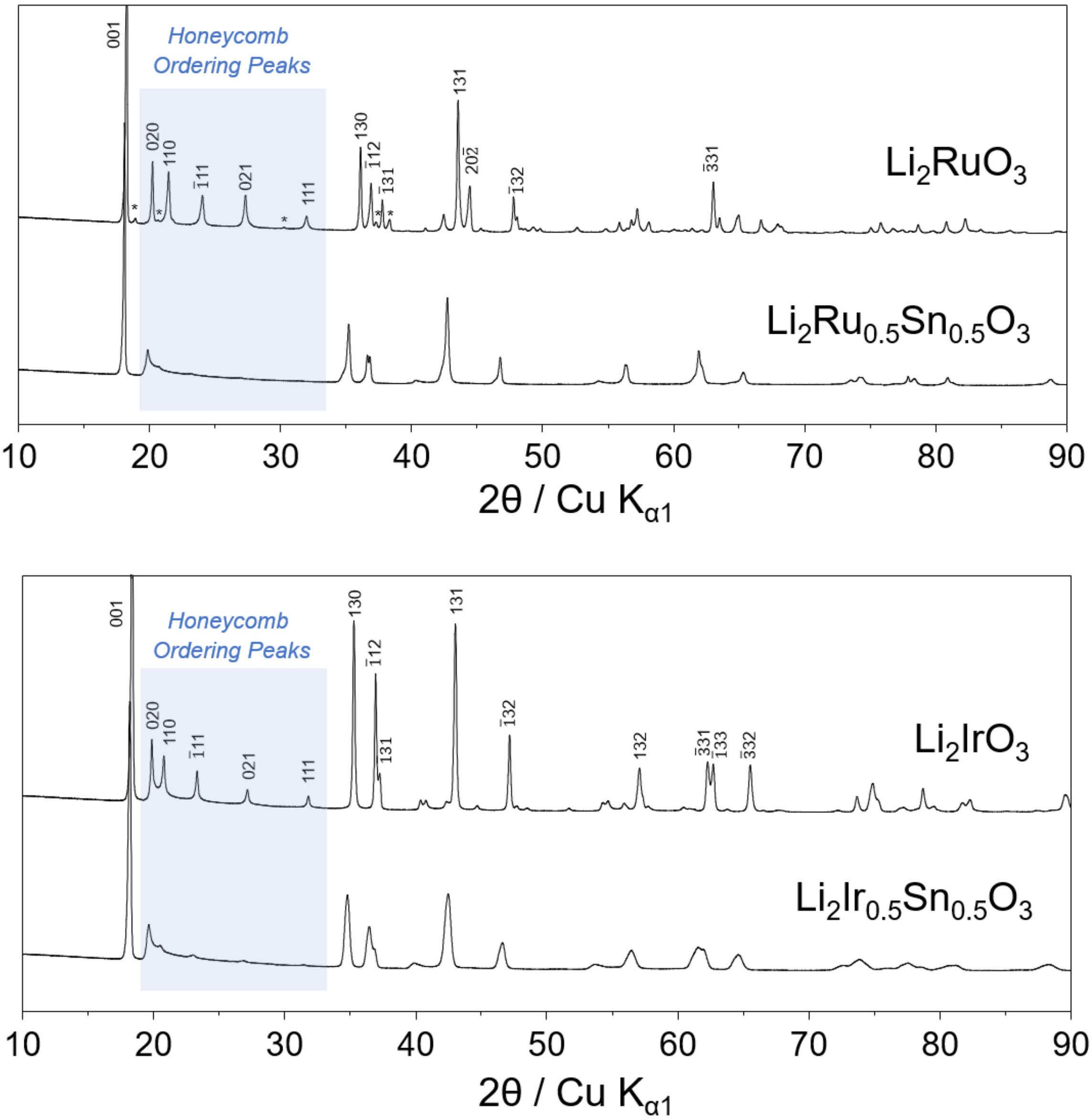

Supplementary Figure 1. Powder X-ray diffraction patterns for the pristine cathode materials indexed to the C2/m space group. The peaks arising from the in-plane honeycomb ordering are highlighted. The superstructure peaks for the Sn-substituted samples exhibit substantial asymmetric peak broadening arising from stacking faults between the honeycomb ordered layers as previously reported. Additional Bragg peaks in the superlattice region labelled* in Li_2RuO_3 arise from slight distortions of Ru to form Ru-Ru dimers as discussed elsewhere.

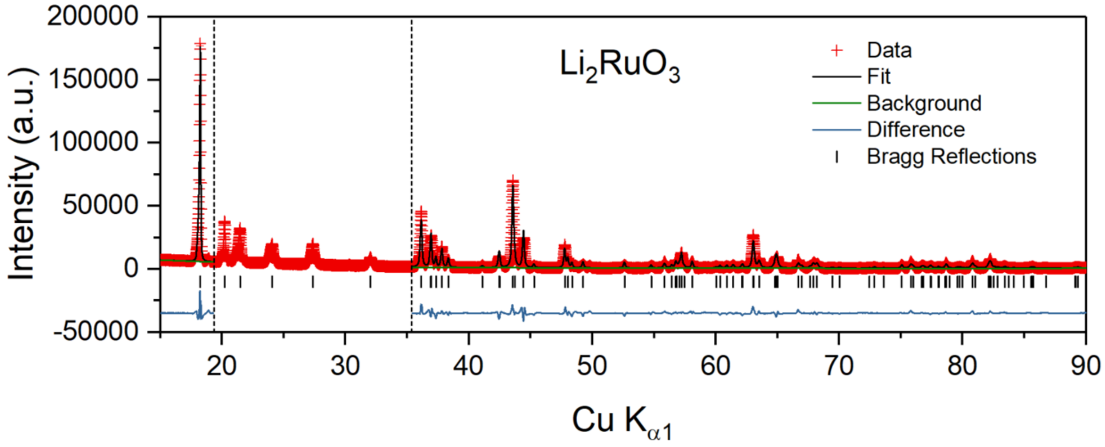

Atom	Wyckoff Position	x	y	z	Occupancy	U_{iso}
Ru	4g	0	0.1721(2)	0	1	0.0187(5)
Li	2b	0	0.5	0	1	0.01
Li	2c	0	0	0.5	1	0.01
Li	4h	0	0.3333	0.5	1	0.01
O	4i	0.223(1)	0	0.237(1)	1	0.01
O	8j	0.255(1)	0.330(1)	0.231(1)	1	0.01
Space Group: C2/m, a = 4.941(1), b = 8.780(1), c = 5.120(1), β = 108.303(2). χ^2 = 9.5						

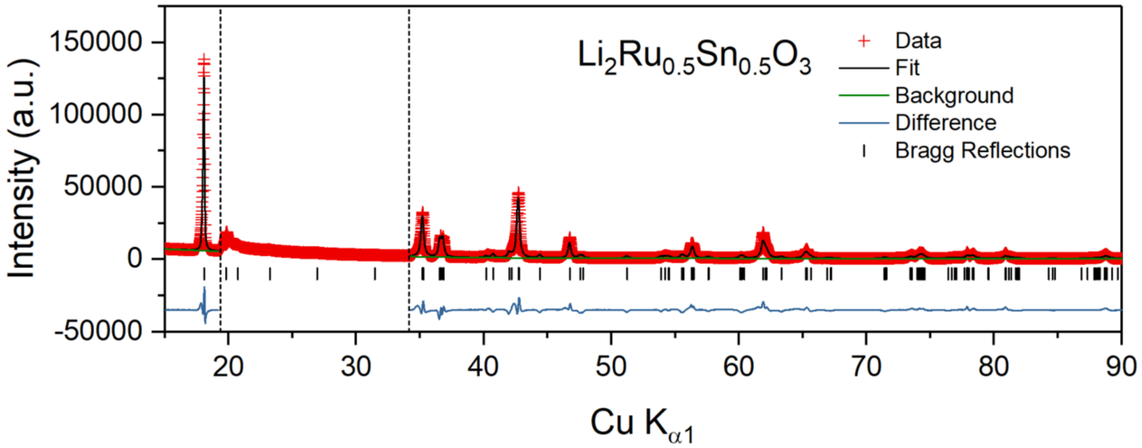

Atom	Wyckoff Position	x	y	z	Occupancy	U_{iso}
Ru / Sn	4g	0	0.1656(3)	0	0.5 / 0.5	0.0282(9)
Li	2b	0	0.5	0	1	0.01
Li	2c	0	0	0.5	1	0.01
Li	4h	0	0.3333	0.5	1	0.01
O	4i	0.185(4)	0	0.206(1)	1	0.01
O	8j	0.221(3)	0.321(2)	0.241(2)	1	0.01
Space Group: C2/m, a = 5.166(4), b = 8.964(1), c = 5.192(4), β = 109.309(1). χ^2 = 12.2						

Supplementary Figure 2. Rietveld Refinements for pristine ruthenates cathodes. Superstructure peaks between 2θ = 18° and 34° have been omitted from the refinement due to the asymmetric peak broadening which conventional rietveld refinement cannot capture.

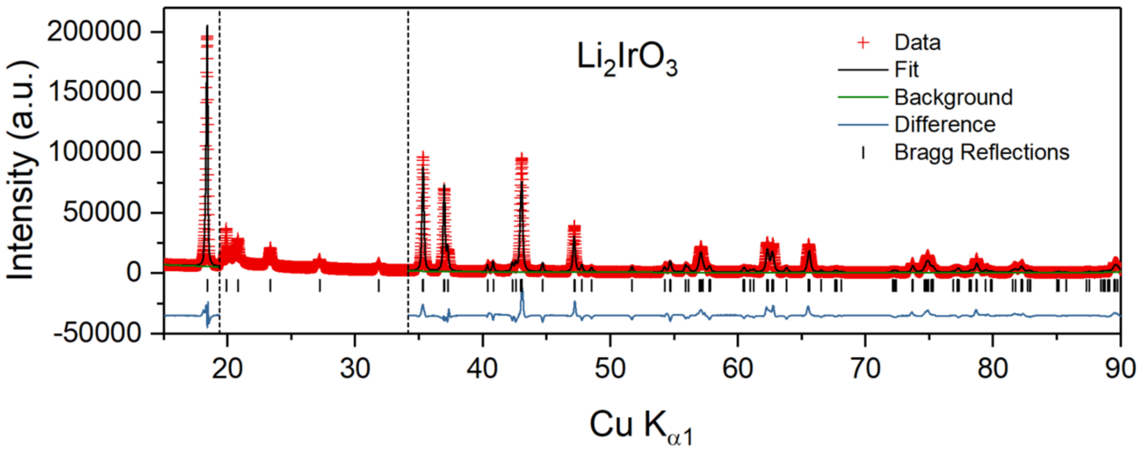

Atom	Wyckoff Position	x	y	z	Occupancy	U_{iso}
Ir	4g	0	0.1650(2)	0	1	0.0197(6)
Li	2b	0	0.5	0	1	0.01
Li	2c	0	0	0.5	1	0.01
Li	4h	0	0.3333	0.5	1	0.01
O	4i	0.168(4)	0	0.190(4)	1	0.01
O	8j	0.230(4)	0.324(2)	0.240(2)	1	0.01
Space Group: C2/m, a = 5.162(2), b = 8.928(1), c = 5.123(2), β = 109.744(5). χ^2 = 15.0						

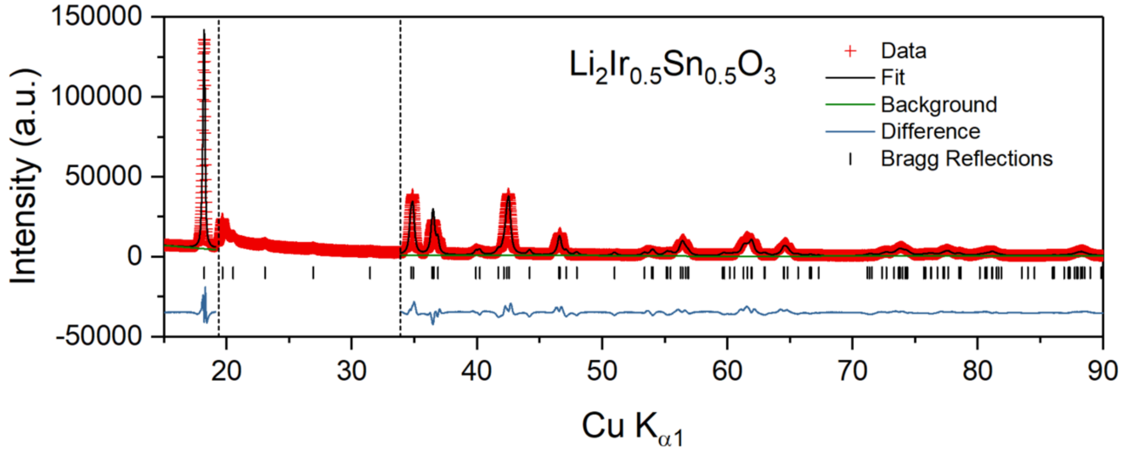

Atom	Wyckoff Position	x	y	z	Occupancy	U_{iso}
Ir / Sn	4g	0	0.1651(3)	0	0.5 / 0.5	0.0207(7)
Li	2b	0	0.5	0	1	0.01
Li	2c	0	0	0.5	1	0.01
Li	4h	0	0.3333	0.5	1	0.01
O	4i	0.167(4)	0	0.189(4)	1	0.01
O	8j	0.227(4)	0.328(2)	0.225(2)	1	0.01
Space Group: C2/m, a = 5.242(4), b = 9.028(1), c = 5.178(4), β = 109.814(1). χ^2 = 13.4						

Supplementary Figure 3. Rietveld Refinements for pristine iridate cathodes. Superstructure peaks between 2θ = 18° and 34° have been omitted from the refinement due to the asymmetric peak broadening which conventional rietveld refinement cannot capture.

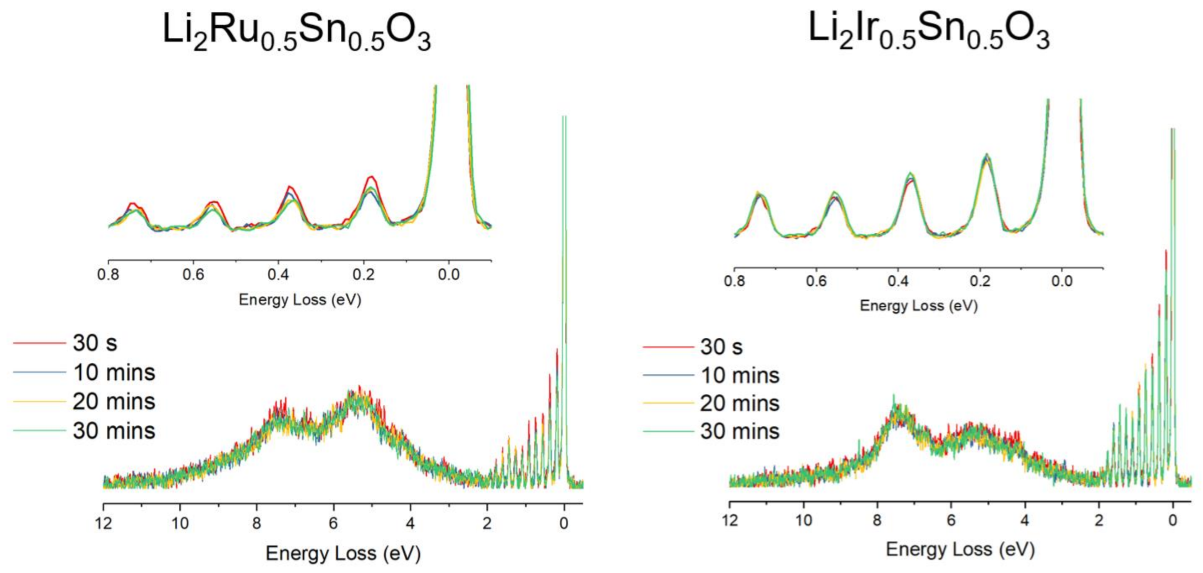

Supplementary Figure 4. RIXS beam sensitivity studies for charged $Li_2Ru_{0.5}Sn_{0.5}O_3$ and $Li_2Ir_{0.5}Sn_{0.5}O_3$ cathode materials measured using an excitation energy of 531 eV at 20K. Spectra were continually acquired every 30s for up to 30 mins at the same sample location. Only a slight decrease in intensity is observed over time for $Li_2Ru_{0.5}Sn_{0.5}O_3$ and there is little change for $Li_2Ir_{0.5}Sn_{0.5}O_3$.
